# African Ancestry Is Associated with Asthma Risk in African Americans

**DOI:** 10.1371/journal.pone.0026807

**Published:** 2012-01-03

**Authors:** Carlos Flores, Shwu-Fan Ma, María Pino-Yanes, Michael S. Wade, Lina Pérez-Méndez, Rick A. Kittles, Deli Wang, Srinivas Papaiahgari, Jean G. Ford, Rajesh Kumar, Joe G. N. Garcia

**Affiliations:** 1 CIBER de Enfermedades Respiratorias, Instituto de Salud Carlos III, Madrid, Spain; 2 Research Unit, Hospital Universitario NS de Candelaria, Tenerife, Spain; 3 Section of Pulmonary and Critical Care Medicine, Department of Medicine, University of Chicago, Chicago, Illinois, United States of America; 4 Institute for Personalized Respiratory Medicine, Department of Medicine, University of Illinois at Chicago, Chicago, Illinois, United States of America; 5 Section of Hematology/Oncology, Department of Medicine, University of Illinois at Chicago, Chicago, Illinois, United States of America; 6 Psychiatry and Behavioral Sciences and Preventive Medicine, Northwestern University, Chicago, Illinois, United States of America; 7 Department of Epidemiology, Johns Hopkins Bloomberg School of Public Health, Baltimore, Maryland, United States of America; 8 Division of Allergy and Immunology, Children's Memorial Hospital and Northwestern University, Chicago, Illinois, United States of America; University of Washington, United States of America

## Abstract

**Background:**

Asthma is a common complex condition with clear racial and ethnic differences in both prevalence and severity. Asthma consultation rates, mortality, and severe symptoms are greatly increased in African descent populations of developed countries. African ancestry has been associated with asthma, total serum IgE and lower pulmonary function in African-admixed populations. To replicate previous findings, here we aimed to examine whether African ancestry was associated with asthma susceptibility in African Americans. In addition, we examined for the first time whether African ancestry was associated with asthma exacerbations.

**Methodology/Principal Findings:**

After filtering for self-reported ancestry and genotype data quality, samples from 1,117 self-reported African-American individuals from New York and Baltimore (394 cases, 481 controls), and Chicago (321 cases followed for asthma exacerbations) were analyzed. Genetic ancestry was estimated based on ancestry informative markers (AIMs) selected for being highly divergent among European and West African populations (95 AIMs for New York and Baltimore, and 66 independent AIMs for Chicago). Among case-control samples, the mean African ancestry was significantly higher in asthmatics than in non-asthmatics (82.0±14.0% vs. 77.8±18.1%, mean difference 4.2% [95% confidence interval (CI):2.0–6.4], *p*<0.0001). This association remained significant after adjusting for potential confounders (odds ratio: 4.55, 95% CI: 1.69–12.29, *p* = 0.003). African ancestry failed to show an association with asthma exacerbations (*p* = 0.965) using a model based on longitudinal data of the number of exacerbations followed over 1.5 years.

**Conclusions/Significance:**

These data replicate previous findings indicating that African ancestry constitutes a risk factor for asthma and suggest that elevated asthma rates in African Americans can be partially attributed to African genetic ancestry.

## Introduction

Asthma is a complex chronic inflammatory condition affected by both genetic and environmental factors [1] that, despite having a low associated mortality (1%), remains a major health risk affecting about 7% (range of 1–18%) of the human population [2]. Among the U.S. population, African descent groups experience higher rates of asthma prevalence and mortality than European Americans [3], [4], suggesting that the disease might be more aggressive in African descent groups [4]. In support of this hypothesis, African Americans with asthma tend to show lower baseline pulmonary function indices [4], greater severity of airway obstruction, increased acuity, and increased hospital admissions compared to European counterparts, despite presenting similar clinical features [5]. Although environmental or cultural differences and socioeconomic factors are thought to be the major determinants of such divergences, accumulated evidence from genetic linkage and association studies suggest that genetic differences among populations may also be involved in ethnic disparities in asthma incidence and severity.

Asthma and atopy-related phenotypes have been linked to incompletely overlapping chromosome regions across the different U.S. ethnic groups [6], [7]. Similarly, genome-wide association studies (GWAS) in asthma in European descent populations have highlighted genes and regions widely associated across independent European samples, such as *GSDMB*-*ORMDL3* [8], *PDE4D* [9], and *CRB1*-*DENND1B* [10], that have been modestly replicated or did not show association in African Americans [11] or Hispanics [12] despite sufficient sample sizes [13]. Conversely, associations with asthma in the *PYHIN1* gene were found to be specific to African descent individuals from North America [13], and the only GWAS in asthma conducted to date in Hispanics revealed novel asthma associations in the *TLE4-CHCHD9* intergenic region with protective alleles likely to be originated in the Native American ancestral population [12]. Although this lack of replicability of regions/genes from linkage analysis and GWAS efforts may reflect inadequate coverage of genetic variation in non-European descent populations in commercial genotyping arrays, these results may also suggest that lack of generalizability of these findings across populations due to allele differences, gene-gene and gene-environmental interactions important for the disease. This scenario would be favored if multiple rare variants, which are more likely to be population specific, are involved in disease causality [14]. This rationale is congruent with current concepts of asthma pathogenesis derived from candidate gene association studies [15], suggesting that no single gene can be considered an asthma susceptibility gene across all populations. Alternatively, ethnic differences for disease incidence and severity may be also explained by the frequency differences for risk alleles underlying the disease [13], [16], which might be substantially increased in specific population groups compared to others [12], [17].

African Americans are a recently admixed population showing, on average, ∼80% admixture with African populations, the remaining being of European ancestry [18]. However, at individual level, African Americans show considerable variation in such proportions [19]. In this situation, for a given disease demonstrating ethnic disparities in predisposition, it is expected that genomic segments that originated from the ancestral ethnic group with the higher risk will be enriched with risk alleles [20]. Thus, a larger proportion of ancestry from the ancestral population with the higher disease risk is expected in affected compared to unaffected individuals. With this premise, previous studies in African-admixed populations have demonstrated that the African genetic ancestry was both associated with risk of asthma and total serum IgE in a Caribbean population [21], and with lower pulmonary function measures in asthmatic and non-asthmatic African Americans [22], [23], the latter suggesting that current diagnosis of severe asthma can be improved if genetic ancestry information is considered for risk assessment [23]. None of these studies have investigated if African ancestry was associated with asthma severity or exacerbations. Furthermore, no study has examined if African ancestry was associated with asthma risk in populations other than the Caribbean. Given the importance of replicating the observations from association studies of genetics with disease [24], here we aimed to determine if the African genetic ancestry was also associated with asthma risk in an independent sample of African Americans. In addition, we examined for the first time whether African ancestry was associated with asthma exacerbations.

## Methods

### Ethics statement

The study was approved by the Institutional Review Boards of Johns Hopkins Bloomberg School of Public Health, Children's Memorial Hospital, John H. Stroger Hospital of Cook County, and Northwestern University Feinberg School of Medicine. All subjects gave written consent.

### Samples from Reducing Emergency Asthma Care in Harlem (REACH)/ Baltimore Asthma Severity Study (BASS)

A total of 875 African Americans, including 394 asthmatic cases and 481 non-asthmatic control subjects, donated a blood sample for genetic analysis in the context of the Reducing Emergency Asthma Care in Harlem (REACH) study and Baltimore Asthma Severity Study (BASS) [25]. These samples have been recently utilized for genetic association studies [11], [26]. Briefly, the participants in both studies responded to a standardized, interviewer-administered questionnaire that included a modified version of the 1987 American Thoracic Society Division of Lung Disease Epidemiology Questionnaire to collect information on demographics, clinical severity, as well as psychosocial determinants of asthma control.

REACH study population consisted of unrelated adults residing in Harlem, a predominantly African American community in New York City, recruited after a visit to the Harlem Hospital Emergency Department (ED) [25]. Briefly, adult English-speaking residents of Central Harlem or West Harlem who visited the Harlem Hospital Center ED for asthma exacerbations or for non-asthmatic and non-allergic conditions between March 1997 and February 1998 were eligible to participate in the study. Participants were asked to visit an outpatient chest clinic with a scheduled interview approximately 3 weeks following the ED visit. Asthma diagnosis was confirmed or discarded based on an evaluation by a trained pulmonary physician and a structured comprehensive asthma questionnaire was administered in a face-to-face interview lasting approximately 1.5 h. Control participants were recruited in the ED at the time of visits for non-respiratory, non-allergic conditions. Control participants reported no history of asthma, allergies or lung disease. Recorded variables included sociodemographic information, self-reported ethnicity, respiratory symptoms (self-reported), family history of asthma, allergy and lung diseases, smoking exposure, alcohol consumption and physical activity, height and weight, utilization of health-care, psychological measures, general health status, use of asthma medication, asthma severity according to the sleep-awakening symptoms in the National Asthma Education and Prevention Program (NAEPP) [27], spirometric values, total IgE and the atopic status. Note, however, that pulmonary function data were obtained only for a small proportion of the asthmatic participants because they were enrolled within less than 6 weeks of requiring emergency care. Atopy was defined by the evidence of allergic sensitization to known allergens, reflected by either a positive skin prick test (SPT; with a wheal diameter 3 mm greater than the saline control) or positive specific IgE (with serum levels >0.35 kUI/mL) to one or more of the following allergens: dust mite, epithelium (cat and dog), ragweed, Timothy grass, *Alternaria* spp and cockroach. The study group was a predominantly low-income population [25].

BASS study population is a community-based convenience sample of unrelated African American adults with residence in Baltimore City, including physician-diagnosed asthmatic cases and control subjects recruited between October 2002 and July 2006. Participants were asked to complete a face-to-face questionnaire. Asthma was defined as a positive response to the question “Have you ever been told by a physician that you have asthma?“ and to a similar question for use of medication for asthma symptoms. Subjects were considered controls based on the negative response to these questions, and a negative spirometry with bronchodilators. Recorded variables for all participants included sociodemographic information, self-reported ethnicity, asthma symptoms (self-reported), family history of asthma, allergy and lung diseases, health-care utilization, smoking exposure, alcohol and drug consumption, psychological measures, and medical history of hypertension, diabetes, depression, heart disease, pneumonia, and kidney diseases. In addition, a spirometric evaluation with bronchodilators was completed for both cases and controls.

### Samples from Chicago Initiative to Raise Asthma Health Equity (CHIRAH) cohort study

The CHIRAH cohort is a community-based longitudinal cohort study of urban children and adults with persistent asthma from Chicago. The cohort was established by a broad community-based screening for households with unrelated persons aged 8–40 with asthma [28], [29]. All eligible study participants were required to have a history of physician-diagnosed persistent, symptomatic asthma, defined in this screening process as requiring at least 8 weeks of asthma medication over the previous 12 months. Recorded information included age, gender, self-reported ancestry, household members with asthma, age at diagnosis, education and income, tobacco exposure, among others [28]. Longitudinal data on asthma exacerbations were also collected at the first visit and at 6 additional follow-up contacts every 3 months. Spirometric assessment was performed only at the first visit following the guidelines of the American Thoracic Society [30]. For the purposes of this study, all 321 asthma patients self reported as African American who provided informed consent to participate in a nested study of genetic risk factors in asthma were included.

### Genotyping

Genotyping was conducted using the iPLEX Gold™ Platform following the manufacturer's protocol (Sequenom, San Diego, CA). Briefly, iPLEX™ assays were scanned by MALDI-TOF mass spectrometry and individual SNP genotype calls were automatically generated using Sequenom TYPER 3.4™ software.

REACH and BASS samples were genotyped for 96 ancestry informative markers (AIMs) selected for being highly divergent (average allele frequency difference, δ, >0.6) among European, African and Native American/Asian populations [26], [31], [32]. Table S1 shows a list of the AIMs and frequency divergences among 60 Europeans and 131 West Africans that were used to derive the ancestry estimates of this study [26]. Kosoy et al [33] showed that subsets of this panel as small as 24 AIMs were useful for ascertaining the origin of subjects from different continents among American populations.

CHIRAH samples were genotyped for an independent panel of 66 highly divergent AIMs among Europeans and Africans (δ≥0.3), which are listed in Table S2 along with frequencies in samples from these populations [34], [35].

Duplicate samples and negative controls were included across the plates to ensure genotyping quality. Genotyping was blinded to the case and control status. The majority of automatic genotype calls (84.5%) had >85% confidence quality scores. All had completion rates above 90% except for rs6894171 in REACH and BASS, for which missing data exceeded 50%. For this reason, this AIM was not considered for further analyses. Samples with genotype calls below 95% were excluded from the study.

### African ancestry estimation

Given that the genetic pool of African Americans is mainly a result of admixture between western African and European populations [18], with limited Native American/Asian genetic influences [36], [37], individual ancestries for all subjects combined were modeled using two ancestral populations (West African and European) by means of STRUCTURE [38]. For ancestry estimation in REACH and BASS, data for 95 AIMs (all but rs6894171) from 60 European and 131 West African samples from our previous studies were used as reference [26]. For ancestry estimation in CHIRAH, data for all 66 AIMs from the HapMap II from 60 unrelated Yoruba Nigerians (YRI) and 60 unrelated Utah residents with ancestry from northern and western Europe (CEU) were used as reference [39]. STRUCTURE was employed under the admixture model using prior population information and independent allele frequencies. The number of parental populations ranged from K = 1 to K = 3 with a burn-in length of 50,000 for 70,000 repetitions. The results showed that the model with best likelihood was K = 2 ancestral populations. For comparison, individual proportions of European and African ancestries were also estimated using ADMIXMAP [40], with 2,000 burn-in and 10,000 iterations, using allele frequencies from parental populations as priors. The diagnosis test for the model (i.e. the test of dispersion) performed by ADMIXMAP program indicated a good fit of the data to the two-parental population admixture model (Bayesian *p*-values>0.35). Pearson and Spearman's rank correlations were used to compare estimates derived from STRUCTURE and ADMIXMAP.

### Statistical analysis

Independent t-tests with equal variances not assumed were initially used to compare the individual ancestry estimates between REACH and BASS asthmatics and controls. We next used multiple logistic regression models to test the association between individual ancestry and asthma, adjusting for relevant covariates using SPSS 19 (SPSS Inc., Chicago, IL). Individual associations of AIMs with asthma were tested by means of the Cochran-Armitage trend test under an additive model and *p*-values obtained by 1,000 permutations by means of a custom script [26]. Logistic regression models were also used to test the association of individual AIMs with asthma, adjusting for the African ancestry estimates.

For the statistical analysis of longitudinal exacerbations in the CHIRAH cohort, exacerbation data (at 3 month intervals up to six times for the number of exacerbations over the preceding 3 months) were modeled as a Poisson regression. All available measurements were included in the analysis using Generalized Estimating Equations (GEE) modeling with robust standard errors, in order to adjust for repeated measures within individuals. For that, the compound symmetry correlation structure was used, assuming that the correlation between a subject's observations was equal across time points. Since GEE adjusted for the co-variances and variances in our model, any over-dispersion (where the mean and the variance were not equal) was also adjusted. The GEE model was fitted using PROC GENMOD in SAS 9.1 (SAS Institute Inc., Cary, NC), with a Poisson distribution and link function of log. In order to determine which covariates to include in the models, we initially included basic demographic covariates, measures of socioeconomic status (SES), including annual household income and insurance status, and well established factors associated with exacerbation, in particular: environmental tobacco exposure, use of inhaled corticosteroids, and baseline forced expiratory volume in one second (FEV_1_). All other potential confounders were included only if associated with the outcome of exacerbation on univariate analysis (*p*-value<0.2). These included the duration of asthma, and asthma severity at baseline (based on the GINA 2002 guidelines algorithm). To test the association between individual ancestry and longitudinal exacerbations, covariates included in the final model were: age, gender, follow-up time, annual household income (categorized as below or above $30,000/year), insurance status (private/non-private), severity at baseline, as determined by application of GINA 2002 guidelines algorithm, baseline FEV_1_, and time varying variables (including smoking status and use of inhaled corticosteroids at each visit). The same model was used for the association of individual AIMs with longitudinal exacerbations, both with and without the African ancestry estimates as a covariate.

In order to judge the significant individual association of AIMs in the context of the multiple comparisons performed, a False Discovery Rate was assessed by QVALUE [41]. A threshold *q*-value of 0.05 was established to declare significance, in order to limit the expected proportion of false positives incurred in the study when a particular individual AIM association test was called significant.

## Results

We excluded samples from individuals self-reporting an ancestry other than African American (5 controls and 12 cases) or with >5% of genotypes missing (37 controls and 25 cases). Thus, the final sample size used for the study consisted on a total of 439 controls and 357 cases (146 controls and 183 cases from REACH study, 293 controls and 174 cases from BASS study) to test the association of African ancestry with asthma susceptibility (see Table S3 for sample characteristics), and 321 cases from CHIRAH study to test the association of African ancestry with asthma exacerbations.

Mean (±SD) African estimates obtained overall case-control samples using STRUCTURE and ADMIXMAP were 79.8±16.5% and 81.5±15.6%, respectively. For REACH, STRUCTURE and ADMIXMAP estimates overall samples were 81.5±15.5% and 83.2±14.3%, respectively. For BASS, STRUCTURE and ADMIXMAP estimates overall samples were 78.5±17.1% and 80.3±14.3%, respectively. For the CHIRAH samples, STRUCTURE and ADMIXMAP estimates were 79.7±11.7% and 81.6±9.7%, respectively. Given that individual African estimates obtained by STRUCTURE and ADMIXMAP were highly correlated across all three studies (Pearson corr. = 0.98, *p*<1.0E-5; Spearman's rho = 0.94, *p*<1.0E-5), in the following we refer only to STRUCTURE estimates.

**Figure 1:**
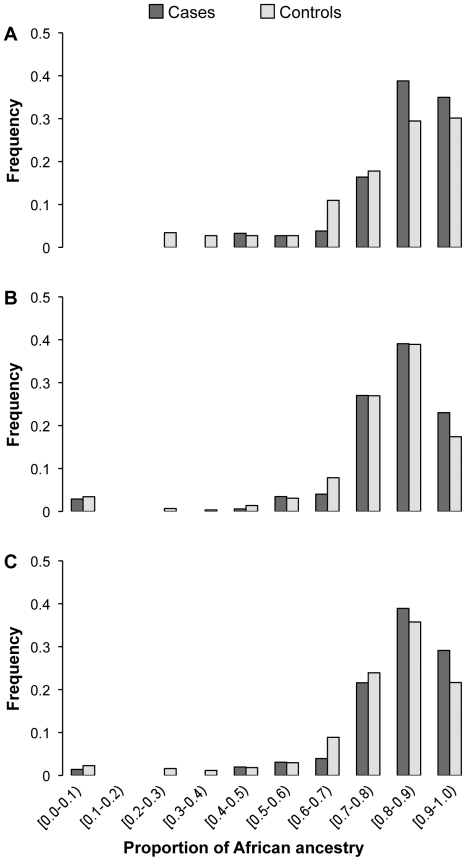
Histograms representing the proportion of individual African ancestry estimates based on 95 AIMs for REACH (A), BASS (B), and overall case-controls (C).

Mean African ancestry was significantly (*p*<0.0001) greater in asthmatics (82.0±14.0%) than in non-asthmatics (77.8±18.1%), with a consistent direction of the effect across the two samples albeit to a minor extent in the community-based study (83.9% vs. 78.3%, *p* = 0.001 for REACH; 80.0% vs. 77.6%, *p* = 0.126 for BASS) (Table 1). The differences between cases and controls were also evident from the distribution of African ancestry proportions by sample group, as represented in Figure 1. To formally test the association of African ancestry with asthma susceptibility, we utilized logistic regression adjusting for age, gender and the study site, and the results again supported a strong association of African ancestry with the risk of asthma (odds ratio [OR]: 4.55; 95% Confidence Interval [CI]: 1.69–12.29; *p* = 0.003) (Table 2). This translated into an OR of 1.16 (95% CI: 1.06–1.28, *p* = 0.002) for the risk of asthma per 10% increase in African ancestry, after adjusting for age, gender and the study site. However, African ancestry did not show a statistically significant association with asthma exacerbations (*p* = 0.965) based on the model of longitudinal data collected for African Americans from the CHIRAH study (exacerbations over the preceding 3 months, 3 month intervals, up to six intervals) (Table 3). Similar results were obtained both for asthma and asthma exacerbations when ADMIXMAP estimates were used in the model (not shown).

**Table 1:** Differences in African ancestry estimates (%, mean±SD) among case-control samples.

Sample	Asthmatics	Non-asthmatics	Difference (95% CI)^a^	*p*-value^b^
Case-control (Ca = 357/Co = 439)	82.0±14.0	77.8±18.1	4.2 (2.0-6.4)	<0.0001
REACH (Ca = 183/Co = 146)	83.9±11.6	78.3±18.8	5.6 (2.2–8.9)	0.001
BASS (Ca = 174/Co = 293)	80.0±15.9	77.6±17.7	2.4 (0.0–5.6)	0.126

a Mean difference between asthmatics and non-asthmatics.

b t-test. Sample sizes in parenthesis.

**Table 2:** Association of African ancestry and asthma using logistic regression models.^a^

Sample	Odds ratio	95% CI	*p*-value
Case-control	4.55	1.69–12.29	0.003
REACH	10.10	2.12–48.21	0.004
BASS	2.62	0.77–8.93	0.125

a Multiple logistic regression models included age, gender and the study site. The African ancestry was introduced as a proportion in the range 0–1.

**Table 3:** Association of demographic, socioeconomic, clinical and genetic factors with asthma exacerbations in CHIRAH study.

Factor	Odds ratio	95% CI	d.f.	Chi-square	Unadjusted *p*-value
African ancestry	1.03	0.28, 3.72	1	0.00	0.965
Age	0.98	0.96, 1.00	1	3.46	0.063
Gender	0.99	0.68, 1.43	1	0.01	0.943
Follow-up time (months)	0.99	0.97, 1.01	1	0.36	0.547
Duration of asthma	1.83	1.19, 2.82	1	6.10	0.014
Corticosteroid use^a^	1.14	0.83, 1.57	1	0.63	0.428
Asthma severity	1.53	1.13, 2.07	1	8.55	0.004
Income	0.84	0.55, 1.27	1	0.71	0.398
Private insurance	0.55	0.38, 0.80	1	7.58	0.006
Tobacco exposure	1.01	0.69, 1.46	1	0.00	0.971
FEV_1_ b (% predicted)	0.82	0.55, 1.24	2	0.90	0.639

a Use at each time point of the model.

b Basal forced expiratory volume in one second.

We might have left unexplained a fraction of individual ancestries assignable to genetic influences from non-African and non-European populations. To test such possibility, we finally analyzed whether ancestry adjustments diminished potentially significant associations between individual AIMs and asthma susceptibility and exacerbations, assuming that they should be due to chance or to the effects of residual population stratification existing in the sample. We first determined whether specific AIMs used for ancestry estimation were associated individually with asthma susceptibility (for simplicity, this was done only for the overall case-control sample) or exacerbations. In case-control samples, twenty-two AIMs were nominally associated in univariate association tests (Table S1). However, after adjusting for relevant covariates, including African ancestry, only four AIMs remained nominally associated, although none of them was considered significant given the number of comparisons performed (lowest *p* = 0.029; lowest *q* = 0.571). Similar results were obtained when testing for individual AIM association with asthma exacerbations in CHIRAH. Five AIMs showed nominal association when ancestry was not considered, and four AIMs remained nominally associated when ancestry was considered in the models, although none of them was considered significant given the number of comparisons performed (lowest *p* = 0.010; lowest *q* = 0.380) (Table S2). Taken together, these results suggest that the adjustment for the ancestry estimates removed the majority of population stratification present in the samples, and that associations of individual AIMs with asthma susceptibility and exacerbations likely represent false positives due to chance. Thus, a two-population admixture model allowed adequately estimating individual ancestries in these African American samples.

## Discussion

Several evidences suggest that genetic risk factors for asthma might differ among populations [6], [7], [11], [13], [42]. However, few studies have focused on the genetic basis of asthma among ethnic minorities such as African Americans, a population with a particularly high incidence of asthma and severe disease. Here we investigated whether genetic ancestry, as estimated from several dozen genetic markers extremely informative for ancestry, was associated with risk of asthma or asthma exacerbations in a total of 1,117 African Americans. We demonstrated that African genetic ancestry associates with asthma risk. This association was not due to potential confounders such as age and gender. In fact, the effect was consistent across the two case-control samples, although statistical significance was not reached for the BASS study. However, confounding effects attributable to other relevant environmental, early life or SES factors, among others, cannot be ruled out. While type I error, biological mechanisms (e.g. due to gene-environment interactions) and/or selection bias may possibly explain the very large difference in effect size observed between the two case-control samples, we favored the latter possibility by incurring in a spectrum of disease bias [43]. Note that all REACH cases were selected for an “extreme” and severe phenotype (as they were recruited from the ED of the Hospital), while recruitment in BASS was community-based, which therefore is expected to have included cases from a broader spectrum of the disease phenotype.

Importantly, our study expands previous observations made for other African-descent populations. Firstly, we replicate the association of African ancestry and risk of asthma in independent African-admixed populations different from those studied previously [21], [22]. Secondly, for the first time, we ascertained the potential effects of African ancestry in asthma exacerbations based on prospective, longitudinal determination of exacerbation phenotypes. In this respect, we admit that this study had limited power to detect modest effects of ancestry in asthma exacerbations (20% for an OR in the 1.20–1.50 range), and that further studies with larger sample sizes will be needed for a better assessment of the risk.

Individuals with self-reported African American descent from the studied locations exhibit ∼80% African ancestry, consistent with previous studies utilizing from >3,000 markers with information for African and European ancestries [20], [22], [44], [45] to 500,000 markers from the genome [19], ensuring that these were properly estimated. Additionally, we used independent sets of 66 and 96 AIMs allowing us to precisely estimate individual ancestral proportions to major continental populations (e.g. the median size of the 90% Bayesian confidence interval overall REACH individuals was 17.0%; P_25_–P_75_: 14.6–18.5%) [33], [46]. Thus, along with the diagnostic results from the two alternative programs used for the admixture model, as well as by judging the limited residual association of individual AIMs after adjusting for the admixture estimates, these data support the precision of the estimated ancestries.

For a case-control study with unrelated individuals, the best source of controls is the population from which cases were ascertained, and recruiting admixed subjects from the same clinic or very nearby geographic location can even minimize differences in the degree of admixture [22]. In the present study, despite obtaining African American case-control samples from individuals residing in similar (urban) areas, and that we were able to find similar effects for the ancestry in asthma risk in two independent case-control samples, we cannot exclude the fact that residual confounding may exist due to unmeasured factors (environmental, social, cultural, or behavioral). In addition, albeit sampling did not include close relative subjects, cryptic relatedness cannot be ruled out from the study. However, simulation studies have evidenced the limited impact of including subjects with close familiar relationships in population-based association studies [47]. Most importantly, we did not have data for the location of residence (as a proxy for exposure to pollution) and SES. Previous studies have not observed African ancestries in African Americans varying with urban or suburban residence [48]. However, it is well known that SES is strongly correlated with race and ethnic background and is a robust predictor of access to and quality of health care and education in the U.S., which, in turn, may be associated with differences in the incidence and outcomes of those diseases [17]. By focusing exclusively on African Americans, we may not totally alleviate this confounding given that skin pigmentation and African ancestry are modestly correlated in African Americans [49]. Earlier studies have suggested that biological endpoints, such as blood pressure, might be influenced by skin color and hypothesized that this was due to SES [50]–[53]. However, a more recent evaluation did not reveal any correlation linking skin pigmentation with health outcomes among African Americans [54]. This makes a residual correlation of individual admixture with social or other environmental agents less likely [55]. In support of this, previous studies exploring the relationship between African ancestry and asthma risk, asthma-related traits or allergic sensitization, did not observe confounding effects due to SES [21], [22], [48].

In summary, a consistent association of African ancestry with asthma risk was observed in a large case-control sample of self-reported African American subjects. Although confounding effects attributable to other relevant risk factors cannot be ruled out, we replicate previous findings and support the notion that ethnic disparities in asthma incidence are affected, in part, by genetic determinants. Frequency differences for risk alleles across populations and/or differential gene-environmental interactions may lead to differential disease susceptibility.

## Supporting Information

Table S1 **Ancestry informative markers used in African American samples from REACH and BASS, and** ***p*** **-values for the association of individual markers with asthma.**
(DOC)

Table S2 **Ancestry informative markers used in African American samples from CHIRAH and** ***p*** **-values for the association of individual markers with asthma exacerbations.**
(DOC)

Table S3 **Relevant demographic and clinical features of REACH and BASS samples.**
(DOC)
