# Supplementary material for: African Ancestry Is Associated with Asthma Risk in African Americans
Source: PLoS One. 2012 Jan 3;7(1):e26807. doi: 10.1371/journal.pone.0026807 (PMC3250386; doi:10.1371/journal.pone.0026807)
Supplement: Table S1 — Ancestry informative markers used in African American samples from REACH and BASS, and p -values for the association of individual markers with asthma. (DOC) [file pone.0026807.s001.doc]

| **Table S1** Ancestry informative markers used in African American samples from REACH and BASS, and *p*-values for the association of individual markers with asthma. | | | | | | | |
| --- | --- | --- | --- | --- | --- | --- | --- |
| rs# | Chromosome | Position^a^ | Freq. European  (n=60) | Freq. W.African  (n=131) | δ (Eur/W.Afr) | *p*-value^b^ | Adjusted *p*-value^c^ |
| rs424436 | 1 | 8005265 | 0.99 | 0.95 | 0.04 | 0.919 | 0.933 |
| rs7504 | 1 | 27110737 | 0.23 | 0.38 | 0.16 | 0.958 | 0.561 |
| rs1931059 | 1 | 35138643 | 0.22 | 0.09 | 0.14 | 0.425 | 0.764 |
| rs10908316 | 1 | 35267397 | 0.88 | 0.08 | 0.80 | 0.108 | 0.842 |
| rs2791966 | 1 | 36087448 | 0.18 | 0.86 | 0.68 | **0.016** | 0.295 |
| rs710232 | 1 | 41975741 | 0.96 | 0.25 | 0.70 | 0.383 | 0.724 |
| rs596985 | 1 | 64049623 | 0.98 | 0.24 | 0.74 | 0.960 | 0.287 |
| rs855833 | 1 | 64130020 | 0.92 | 0.11 | 0.81 | 0.488 | 0.057 |
| rs17035850 | 1 | 116833545 | 0.97 | 0.15 | 0.82 | **0.001** | 0.249 |
| rs6695965 | 1 | 145526715 | 0.86 | 0.12 | 0.74 | **0.028** | 0.511 |
| rs2274533 | 1 | 146662406 | 0.23 | 0.48 | 0.25 | 0.862 | 0.759 |
| rs2814778 | 1 | 157441307 | 0.99 | 0.00 | 0.99 | 0.176 | 0.225 |
| rs12074150 | 1 | 171883530 | 1.00 | 0.35 | 0.64 | 0.420 | 0.859 |
| rs2065160 | 1 | 203057600 | 0.87 | 0.54 | 0.33 | 0.978 | 0.333 |
| rs6604611 | 1 | 214721874 | 0.10 | 0.87 | 0.77 | 0.065 | 0.824 |
| rs6698938 | 1 | 228051979 | 0.99 | 0.25 | 0.74 | 0.214 | 0.894 |
| rs2502342 | 1 | 241137090 | 0.06 | 0.73 | 0.66 | 0.106 | 0.442 |
| rs883399 | 2 | 9551551 | 0.60 | 0.38 | 0.22 | 0.613 | 0.752 |
| rs300152 | 2 | 17850164 | 0.19 | 0.24 | 0.05 | 0.384 | 0.204 |
| rs11124405 | 2 | 35041515 | 0.93 | 0.14 | 0.79 | **0.038** | 0.539 |
| rs13385952 | 2 | 41384902 | 0.84 | 0.06 | 0.78 | 0.320 | 0.873 |
| rs1881244 | 2 | 73498597 | 0.07 | 0.87 | 0.80 | **0.020** | 0.470 |
| rs12714168 | 2 | 86184858 | 0.86 | 0.11 | 0.75 | **0.002** | 0.207 |
| rs6576989 | 2 | 96888826 | 0.90 | 0.09 | 0.82 | 0.111 | 0.844 |
| rs260714 | 2 | 108928927 | 0.13 | 0.83 | 0.70 | 0.346 | 0.835 |
| rs951954 | 2 | 109816794 | 0.01 | 0.79 | 0.78 | 0.073 | 0.524 |
| rs901304 | 2 | 163124608 | 0.78 | 0.15 | 0.63 | 0.085 | 0.387 |
| rs6748661 | 2 | 195391085 | 0.18 | 0.88 | 0.70 | 0.494 | 0.588 |
| rs11713766 | 3 | 373089 | 0.02 | 0.83 | 0.81 | 0.068 | 0.540 |
| rs2470644 | 3 | 5751857 | 0.92 | 0.22 | 0.70 | 0.828 | 0.152 |
| rs2197896 | 3 | 30007534 | 0.95 | 0.12 | 0.83 | 0.105 | 0.811 |
| rs9311121 | 3 | 35903067 | 0.05 | 0.87 | 0.82 | **0.004** | 0.145 |
| rs13069719 | 3 | 71589235 | 0.20 | 0.23 | 0.03 | 0.176 | 0.182 |
| rs2660769 | 3 | 87163186 | 0.72 | 0.01 | 0.71 | **0.028** | 0.497 |
| rs12489482 | 3 | 106062098 | 0.15 | 0.86 | 0.71 | **0.041** | 0.425 |
| rs6437783 | 3 | 109655507 | 0.83 | 0.72 | 0.11 | 0.186 | 0.272 |
| rs11714866 | 3 | 111550592 | 0.91 | 0.20 | 0.71 | 0.100 | 0.469 |
| rs6772085 | 3 | 120067253 | 0.93 | 0.19 | 0.74 | **0.050** | 0.788 |
| rs2165139 | 3 | 140697160 | 0.89 | 0.96 | 0.07 | 0.091 | 0.312 |
| rs6439896 | 3 | 141347043 | 0.97 | 0.34 | 0.63 | 0.707 | 0.437 |
| rs1439013 | 3 | 154064681 | 0.92 | 0.25 | 0.67 | 0.351 | **0.045** |
| rs9290363 | 3 | 170477801 | 0.08 | 0.88 | 0.80 | **0.013** | 0.264 |
| rs10032047 | 4 | 63410428 | 0.04 | 0.94 | 0.90 | 0.067 | 0.777 |
| rs7689609 | 4 | 72302238 | 0.84 | 0.00 | 0.84 | 0.660 | 0.058 |
| rs7687935 | 4 | 82284590 | 0.80 | 0.20 | 0.60 | **0.033** | 0.439 |
| rs7662047 | 4 | 103310753 | 0.03 | 0.89 | 0.86 | 0.369 | 0.846 |
| rs7657799 | 4 | 105594872 | 0.97 | 0.21 | 0.76 | 0.222 | 0.124 |
| rs13108157 | 4 | 151750213 | 0.12 | 0.94 | 0.82 | 0.919 | **0.042** |
| rs6829588 | 4 | 165441421 | 0.17 | 0.81 | 0.65 | **0.017** | 0.303 |
| rs2332031 | 4 | 171979533 | 0.88 | 0.12 | 0.76 | 0.947 | 0.100 |
| rs814597 | 5 | 10521929 | 0.12 | 0.17 | 0.05 | 0.428 | 0.586 |
| rs463240 | 5 | 25880903 | 0.05 | 0.78 | 0.73 | 0.398 | 0.512 |
| rs35395 | 5 | 33984346 | 0.06 | 0.78 | 0.72 | 0.270 | 0.462 |
| rs16891982 | 5 | 33987450 | 0.09 | 1.00 | 0.91 | **0.028** | 0.837 |
| rs10059859 | 5 | 59267064 | 0.14 | 0.81 | 0.67 | 0.105 | 0.983 |
| rs6894171 | 5 | 72798481 | 0.06 | 0.88 | 0.82 | NA | NA |
| rs1443985 | 5 | 119453406 | 0.87 | 0.13 | 0.74 | 0.095 | 0.694 |
| rs4513684 | 5 | 147632278 | 0.98 | 0.21 | 0.77 | 0.281 | 0.907 |
| rs1551765 | 5 | 153156771 | 0.17 | 0.29 | 0.12 | 0.697 | 0.656 |
| rs567442 | 5 | 176185891 | 0.93 | 0.13 | 0.80 | **0.031** | 0.377 |
| rs6909271 | 6 | 143379 | 0.17 | 0.91 | 0.74 | 0.962 | 0.232 |
| rs6459548 | 6 | 17590296 | 0.01 | 0.79 | 0.78 | **0.042** | 0.400 |
| rs1341567 | 6 | 76684896 | 0.98 | 0.19 | 0.79 | **0.012** | 0.314 |
| rs2497150 | 6 | 84903732 | 0.07 | 0.82 | 0.75 | **0.007** | 0.103 |
| rs794672 | 6 | 95515038 | 0.12 | 0.95 | 0.83 | 0.177 | 0.743 |
| rs218867 | 6 | 121440234 | 0.80 | 0.12 | 0.68 | 0.100 | 0.863 |
| rs6930928 | 6 | 156647248 | 0.07 | 0.85 | 0.78 | **0.020** | 0.317 |
| rs7810554 | 7 | 15107715 | 0.80 | 0.01 | 0.79 | **0.005** | 0.094 |
| rs7784684 | 7 | 40134287 | 0.02 | 0.87 | 0.85 | 0.359 | 0.841 |
| rs10264353 | 7 | 43287602 | 0.77 | 0.00 | 0.77 | 0.249 | 0.615 |
| rs10257477 | 7 | 107491924 | 0.98 | 0.28 | 0.70 | 0.544 | 0.593 |
| rs3094537 | 7 | 109341905 | 0.96 | 0.18 | 0.78 | 0.484 | **0.049** |
| rs2021782 | 7 | 131785535 | 0.89 | 0.12 | 0.77 | 0.253 | 0.285 |
| rs10954631 | 7 | 138190166 | 0.10 | 0.85 | 0.75 | 0.528 | 0.199 |
| rs6601288 | 8 | 8980840 | 0.30 | 0.13 | 0.17 | 0.364 | 0.363 |
| rs11778591 | 8 | 12764720 | 0.89 | 0.33 | 0.56 | **0.017** | 0.191 |
| rs2439522 | 8 | 97602942 | 0.86 | 0.96 | 0.10 | 0.776 | 0.936 |
| rs12347078 | 9 | 334508 | 0.95 | 0.12 | 0.83 | 0.265 | 0.529 |
| rs4478653 | 9 | 21843221 | 0.59 | 0.18 | 0.41 | 0.695 | 0.369 |
| rs587364 | 9 | 124800684 | 0.14 | 1.00 | 0.86 | 0.971 | 0.130 |
| rs10748592 | 10 | 94868345 | 0.24 | 0.39 | 0.15 | 0.827 | 0.905 |
| rs1572396 | 10 | 117315011 | 0.29 | 0.62 | 0.33 | 0.884 | 0.280 |
| rs6485600 | 11 | 12216278 | 0.31 | 0.66 | 0.35 | 0.073 | 0.123 |
| rs1638567 | 11 | 66881799 | 0.92 | 0.15 | 0.77 | 0.506 | 0.537 |
| rs2458640 | 11 | 77713504 | 0.77 | 0.06 | 0.72 | 0.390 | **0.029** |
| rs533571 | 11 | 100355412 | 0.30 | 0.16 | 0.14 | 0.200 | 0.743 |
| rs4936512 | 11 | 119660546 | 0.79 | 0.69 | 0.10 | 0.735 | 0.809 |
| rs1648180 | 11 | 127554612 | 0.30 | 0.44 | 0.14 | 0.440 | 0.177 |
| rs2293048 | 12 | 116149208 | 0.13 | 0.56 | 0.43 | 0.267 | 0.733 |
| rs1540979 | 13 | 93888693 | 0.85 | 0.96 | 0.12 | 0.110 | 0.305 |
| rs730570 | 14 | 100212643 | 0.83 | 0.17 | 0.65 | 0.915 | 0.273 |
| rs1129038 | 15 | 26030454 | 0.69 | 0.00 | 0.69 | 0.534 | 0.677 |
| rs1426654 | 15 | 46213776 | 0.99 | 0.01 | 0.98 | 0.419 | 0.526 |
| rs11073967 | 15 | 89366808 | 0.59 | 0.01 | 0.58 | 0.392 | 0.992 |
| rs9937955 | 16 | 10858027 | 0.24 | 0.57 | 0.33 | **0.017** | 0.060 |
| rs1557519 | 16 | 14158804 | 0.90 | 0.04 | 0.86 | 0.150 | 0.834 |

^a^From NCBI build 36.3; ^b^Cochran-Armitage trend test for the association of individual markers with asthma; ^c^Logistic regression for the association of individual markers with asthma adjusting for age, gender, location of residence and West African ancestry as estimated by STRUCTURE. *p*-values ≤ 0.05 are shown in bold. NA, not analyzed due to the excess of missing data.
