# Supplementary material for: African Ancestry Is Associated with Asthma Risk in African Americans
Source: PLoS One. 2012 Jan 3;7(1):e26807. doi: 10.1371/journal.pone.0026807 (PMC3250386; doi:10.1371/journal.pone.0026807)
Supplement: Table S2 — Ancestry informative markers used in African American samples from CHIRAH and p -values for the association of individual markers with asthma exacerbations. (DOC) [file pone.0026807.s002.doc]

| **Table S2** Ancestry informative markers used in African American samples from CHIRAH and *p*-values for the association of individual markers with asthma exacerbations. | | | | | | | |
| --- | --- | --- | --- | --- | --- | --- | --- |
| rs # | Chromosome | Position^a^ | Freq. European  (n=60) | Freq. W.African  (n=60) | δ (Eur/W.Afr) | *p*-value^b^ | Adjusted *p*-value^c^ |
| rs1934393 | 1 | 48981205 | 0.15 | 0.78 | 0.63 | **0.050** | 0.052 |
| rs2592888 | 1 | 157852197 | 0.92 | 0.12 | 0.80 | 0.528 | 0.580 |
| rs2817611 | 1 | 11535750 | 0.96 | 0.29 | 0.67 | 0.296 | 0.282 |
| rs3806218 | 1 | 145478407 | 0.39 | 0.93 | 0.54 | 0.922 | 0.985 |
| rs6684063 | 1 | 30471927 | 0.82 | 0.24 | 0.58 | 0.950 | 0.941 |
| rs7535375 | 1 | 233979782 | 0.59 | 0.11 | 0.48 | 0.478 | 0.459 |
| rs868179 | 2 | 177257743 | 0.93 | 0.30 | 0.63 | **0.030** | **0.026** |
| rs1036543 | 2 | 133392684 | 0.98 | 0.34 | 0.64 | 0.722 | 0.691 |
| rs1470524 | 2 | 44983019 | 0.80 | 0.18 | 0.62 | 0.917 | 0.951 |
| rs3860446 | 2 | 103855783 | 0.73 | 0.02 | 0.71 | 0.758 | 0.772 |
| rs4852696 | 2 | 83005152 | 0.19 | 0.86 | 0.67 | 0.475 | 0.440 |
| rs10498255 | 2 | 231320474 | 0.81 | 0.21 | 0.60 | 0.099 | 0.108 |
| rs304051 | 3 | 4553306 | 0.67 | 0.18 | 0.49 | 0.962 | 0.974 |
| rs1395771 | 3 | 97946270 | 0.97 | 0.36 | 0.61 | 0.287 | 0.368 |
| rs1984473 | 3 | 157293978 | 0.43 | 0.94 | 0.51 | 0.430 | 0.442 |
| rs6804094 | 3 | 188540664 | 0.63 | 0.98 | 0.35 | 0.630 | 0.664 |
| rs9310888 | 3 | 29261766 | 0.94 | 0.47 | 0.47 | 0.803 | 0.801 |
| rs1398829 | 4 | 21632373 | 0.03 | 0.74 | 0.71 | 0.522 | 0.512 |
| rs9307613 | 4 | 130576854 | 0.56 | 0.13 | 0.43 | 0.095 | 0.151 |
| rs10519979 | 4 | 149854401 | 0.45 | 0.15 | 0.30 | 0.275 | 0.279 |
| rs153898 | 5 | 94214378 | 0.29 | 0.91 | 0.62 | 0.121 | 0.123 |
| rs257748 | 5 | 15872615 | 0.34 | 0.68 | 0.34 | 0.175 | 0.179 |
| rs1477277 | 5 | 180607628 | 0.77 | 0.03 | 0.74 | 0.422 | 0.419 |
| rs6883095 | 5 | 79926803 | 0.42 | 0.84 | 0.42 | 0.132 | 0.139 |
| rs9292118 | 5 | 55935953 | 0.70 | 0.24 | 0.46 | 0.310 | 0.348 |
| rs10515535 | 5 | 143496335 | 0.43 | 0.96 | 0.53 | 0.188 | 0.223 |
| rs993314 | 6 | 73495293 | 0.84 | 0.24 | 0.60 | 0.931 | 0.937 |
| rs6569792 | 6 | 132736444 | 0.79 | 0.08 | 0.71 | 0.156 | 0.148 |
| rs6911727 | 6 | 9061397 | 0.63 | 0.16 | 0.47 | 0.605 | 0.607 |
| rs9320808 | 6 | 121696295 | 0.15 | 0.86 | 0.71 | **0.009** | **0.010** |
| rs10484578 | 6 | 35354297 | 0.37 | 1.00 | 0.63 | 0.148 | 0.132 |
| rs802524 | 7 | 145582575 | 0.97 | 0.17 | 0.80 | 0.576 | 0.623 |
| rs10214949 | 7 | 78886529 | 0.83 | 0.43 | 0.40 | 0.701 | 0.670 |
| rs10248051 | 7 | 51086847 | 0.76 | 0.12 | 0.64 | 0.634 | 0.619 |
| rs1898280 | 8 | 116143636 | 0.88 | 0.15 | 0.73 | **0.033** | **0.035** |
| rs7463344 | 8 | 33983069 | 0.01 | 0.49 | 0.48 | 0.300 | 0.300 |
| rs9325872 | 8 | 20524551 | 0.34 | 0.81 | 0.47 | 0.234 | 0.227 |
| rs2840290 | 9 | 16723957 | 0.76 | 0.21 | 0.55 | 0.900 | 0.885 |
| rs4013967 | 9 | 76086890 | 0.23 | 0.68 | 0.45 | 0.177 | 0.181 |
| rs10491654 | 9 | 101179348 | 0.29 | 0.61 | 0.32 | 0.692 | 0.712 |
| rs1397618 | 10 | 120822665 | 0.02 | 0.50 | 0.48 | 0.054 | 0.052 |
| rs2785279 | 10 | 33749882 | 0.86 | 0.13 | 0.73 | 0.587 | 0.544 |
| rs879780 | 11 | 129513314 | 0.91 | 0.18 | 0.73 | 0.257 | 0.278 |
| rs948360 | 11 | 65863301 | 0.10 | 0.71 | 0.61 | 0.472 | 0.513 |
| rs10501474 | 11 | 80078295 | 0.34 | 0.86 | 0.52 | 0.557 | 0.562 |
| rs249847 | 12 | 97391847 | 0.53 | 0.08 | 0.45 | 0.484 | 0.470 |
| rs4034627 | 12 | 126963425 | 0.10 | 0.71 | 0.61 | 0.263 | 0.299 |
| rs4076700 | 12 | 115867703 | 0.17 | 0.77 | 0.60 | 0.075 | 0.084 |
| rs4762106 | 12 | 64304740 | 0.19 | 0.83 | 0.64 | 0.698 | 0.724 |
| rs10506816 | 12 | 78448988 | 0.99 | 0.28 | 0.71 | 0.615 | 0.618 |
| rs5000507 | 13 | 80986955 | 0.70 | 0.08 | 0.62 | 0.848 | 0.810 |
| rs10492585 | 13 | 104184177 | 0.96 | 0.03 | 0.93 | 0.112 | 0.127 |
| rs2296274 | 14 | 60986931 | 0.26 | 0.98 | 0.72 | 0.507 | 0.541 |
| rs9323178 | 14 | 22183486 | 0.52 | 0.12 | 0.40 | 0.686 | 0.670 |
| rs10131076 | 14 | 79844138 | 0.89 | 0.39 | 0.50 | 0.659 | 0.704 |
| rs9302185 | 15 | 52742156 | 0.17 | 0.89 | 0.72 | 0.607 | 0.573 |
| rs10520678 | 15 | 86738287 | 0.76 | 0.15 | 0.61 | 0.367 | 0.351 |
| rs30125 | 16 | 14262162 | 0.88 | 0.51 | 0.37 | 0.307 | 0.318 |
| rs4130513 | 16 | 77016251 | 0.95 | 0.21 | 0.74 | 0.206 | 0.213 |
| rs10491097 | 17 | 19301803 | 0.30 | 0.98 | 0.68 | 0.104 | 0.142 |
| rs1013459 | 18 | 11690534 | 0.89 | 0.23 | 0.66 | **0.027** | **0.025** |
| rs12953952 | 18 | 65888907 | 0.07 | 0.93 | 0.86 | 0.639 | 0.593 |
| rs888861 | 19 | 40073692 | 0.61 | 0.06 | 0.55 | 0.089 | 0.093 |
| rs354747 | 20 | 58346055 | 0.63 | 0.06 | 0.57 | 0.990 | 0.965 |
| rs708915 | 20 | 8348667 | 0.83 | 0.25 | 0.58 | 0.814 | 0.808 |
| rs138022 | 22 | 38942982 | 0.26 | 0.88 | 0.62 | 0.547 | 0.582 |

^a^From NCBI build 36.3; ^b^For the association of individual markers with longitudinal exacerbations; ^c^West African ancestry, as estimated by STRUCTURE, included as a covariate in the longitudinal exacerbations model. *p*-values ≤ 0.05 are shown in bold.
