# Supplementary material for: African Ancestry Is Associated with Asthma Risk in African Americans
Source: PLoS One. 2012 Jan 3;7(1):e26807. doi: 10.1371/journal.pone.0026807 (PMC3250386; doi:10.1371/journal.pone.0026807)
Supplement: Table S3 — Relevant demographic and clinical features of REACH and BASS samples. (DOC) [file pone.0026807.s003.doc]

| **Table S3** Relevant demographic and clinical features of REACH and BASS samples. | | | | | | |
| --- | --- | --- | --- | --- | --- | --- |
|  | REACH | | | BASS | | |
| Variable | Cases (183) | Controls (146) | *p*-value | Cases (174) | Controls (293) | *p*-value |
| Age (years), mean±SD | 41.9±13.5 (183) | 37.3±12.1 (78) | 0.011^a^ | 29.6±7.7 (174) | 30.5±7.2 (293) | 0.174^a^ |
| Gender (% females) | 61.7 (183) | 46.6 (146) | 0.006^b^ | 67.8 (174) | 47.4 (293) | <0.001^b^ |
| FEV_1_^c^ (% predicted), mean±SD | 64.5±25.5 (76) | NA | NA | 88.5±22.2 (158) | 99.2±14.6 (267) | <0.001^a^ |
| Atopy (% of positives) | 80.8 (146) | NA | NA | NA | NA | NA |
| Serum IgE (kUI/mL), mean±SD | 182.0±4.8 (183) | NA | NA | NA | NA | NA |
| Nighttime severity (% with ≥2 episodes/week) | 72.9 (181) | NA | NA | NA | NA | NA |
| Asthma attacks (% with >4 episodes/month) | 52.3 (174) | NA | NA | NA | NA | NA |
| ^a^t-test; ^b^χ^2^ test; ^c^Basal forced expiratory volume in one second. In parentheses, total number of samples with available data. NA, not analyzed. | | | | | | |
